# Assessment of cerebrovascular function in patients with sickle cell disease using transfer function analysis

**DOI:** 10.14814/phy2.15472

**Published:** 2022-10-05

**Authors:** Ece Su Sayin, Olivia Sobczyk, Julien Poublanc, David J. Mikulis, Joseph A. Fisher, Kevin H. M. Kuo, James Duffin

**Affiliations:** ^1^ Department of Physiology University of Toronto Toronto Canada; ^2^ Departments of Anaesthesia and Pain Management University Health Network Toronto Canada; ^3^ Joint Department of Medical Imaging and the Functional Neuroimaging Laboratory University Health Network Toronto Canada; ^4^ Institute of Medical Sciences University of Toronto Toronto Canada; ^5^ Division of Medical Oncology and Hematology, Department of Medicine University of Toronto Toronto Ontario Canada

**Keywords:** cerebrovascular reactivity, CO_2_, sickle cell disease, transfer function analysis

## Abstract

In patients with sickle cell disease (SCD), the delivery of oxygen to the brain is compromised by anemia, abnormal rheology, and steno‐occlusive vascular disease. Successful compensation depends on an increase in oxygen supply such as that provided by an increase in cerebral blood flow (CBF). We used magnetic resonance imaging to provide a high‐resolution assessment of the ability of SCD patients to respond to a vasoactive stimulus in middle, anterior, and posterior cerebral artery territories for both white and gray matter. Cerebrovascular reactivity (CVR) was measured as the blood oxygen level dependent signal (a surrogate for CBF) response to an increase in the end tidal partial pressure of CO_2_ (P_ET_CO_2_). The dynamic aspect of the response was measured as the time constant of the first order response kinetics (tau). To confirm and support these findings we used an alternative examination of the response, transfer function analysis (TFA), to measure the responsiveness (gain), the speed of response (phase), and the consistency of the response over time (coherence). We tested 34 patients with SCD and compared the results to those of 24 healthy controls participants. The results from a three‐way ANOVA showed that patients with SCD have reduced CVR (*p* < 0.001) and lower coherence (*p* < 0.001) in gray matter and white matter and reduced gain in gray matter only (*p* < 0.001). In terms of the speed of the response to CO_2_, tau (*p* < 0.001) and TFA phase (*p* < 0.001) were increased in SCD patients compared to healthy control subjects. These findings show that the cerebrovascular responsiveness to CO_2_ in patients with SCD is both decreased and slowed compared to healthy controls.

## Graphical abstract

We used magnetic resonance imaging to provide a high‐resolution assessment of the ability of sickle cell disease patients to respond to a vasoactive stimulus in middle, anterior and posterior cerebral artery territories for both white and grey matter. Our findings show that the cerebrovascular responsiveness to carbon dioxide in these patients is both decreased and slowed compared to healthy controls.

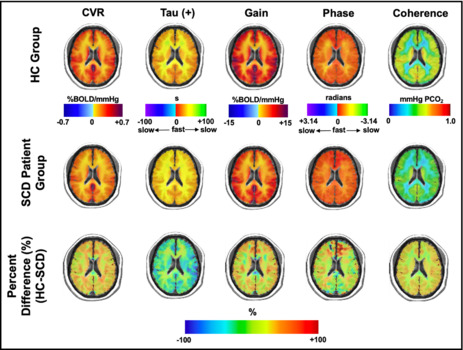

## 1 INTRODUCTION

Sickle cell disease (SCD) is a hereditary red blood cell disorder characterized by hemolysis and vaso‐occlusion resulting in end‐organ complications and early mortality. Patients with SCD are at very high risk of cerebral infarction, hemorrhage, and vasculopathy (Bernaudin et al., 2011; Pegelow et al., 2002).

The exact pathophysiology of neurologic complications in patients with SCD has not been fully elucidated. Compensatory mechanisms are recruited to maintain tissue oxygenation in face of altered oxygen affinity and vasculopathic damages. An increase in cerebral blood flow (CBF) roughly proportional a reduction in oxygen carrying capacity acts to restore brain oxygen delivery (Fields et al., 2018), accomplished in part by vascular remodeling to increased diameters of larger vessels (Liu et al., 2014; National Institutes of Health, 2014). It is likely that in vulnerable locations such as watershed areas of the brain, the compensation is incomplete resulting in increased oxygen extraction fractions (OEF) (Fields et al., 2018), which may, at least on occasion, fail to provide the brain oxygen consumption needed for continuing tissue viability, resulting in multiple tissue infarctions (Fields et al., 2018; Guilliams et al., 2017). The locations of the infarctions are predominantly in the watershed areas, but not otherwise associated with detectable localized vascular abnormalities, leaving inadequate CBF regulation as a likely etiologic factor. Trans‐cranial Doppler (TCD) monitoring middle cerebral artery blood velocity (MCAv) indicates the relationship between high flow velocities and stroke (Adams et al., 1992), and a reduction in MCAv following blood transfusion accompanied by a reduced risk of stroke (Adams et al., 1998; Adams & Brambilla, 2005). However, the relationship between increased blood velocity in a large artery, its flow, flow in watershed areas, flow response to increase demand, reasons for periodic inadequate supply, and pathophysiology of stroke are not currently understood (Prohovnik et al., 2009).

Here we focus on measures of CBF regulation. Research studies have investigated characteristic values for CBF, OEF, and the cerebral metabolic rate of oxygen consumption in patients with SCD compared to healthy controls (Fields et al., 2018; Guilliams et al., 2017). Oxygen extraction fraction in patients with SCD are mixed in this area, with some studies indicating normal OEF (Bush et al., 2014; Li et al., 2020), and others indicating higher (Fields et al., 2018; Jordan et al., 2016) and lower OEF (Vaclavu et al., 2020). All these measures are performed with the subject in a basal state at rest, but it is possible to explore the changes in vascular tone to a vasoactive stimulus, example, using hypercapnia. Reduced vasodilatory reserve has been documented in adult patients with SCD (Sayin et al., 2022) and children with SCD (Kosinski et al., 2017; Leung, Duffin, et al., 2016). Employing transfer function analysis (TFA) provides an alternative analysis of the response to hypercapnia measured as CVR, enabling a more rigorous comparison to responses in healthy children (Leung, Kosinski, et al., 2016). However, TFA has not been studied in adults with SCD.

**FIGURE 1:**
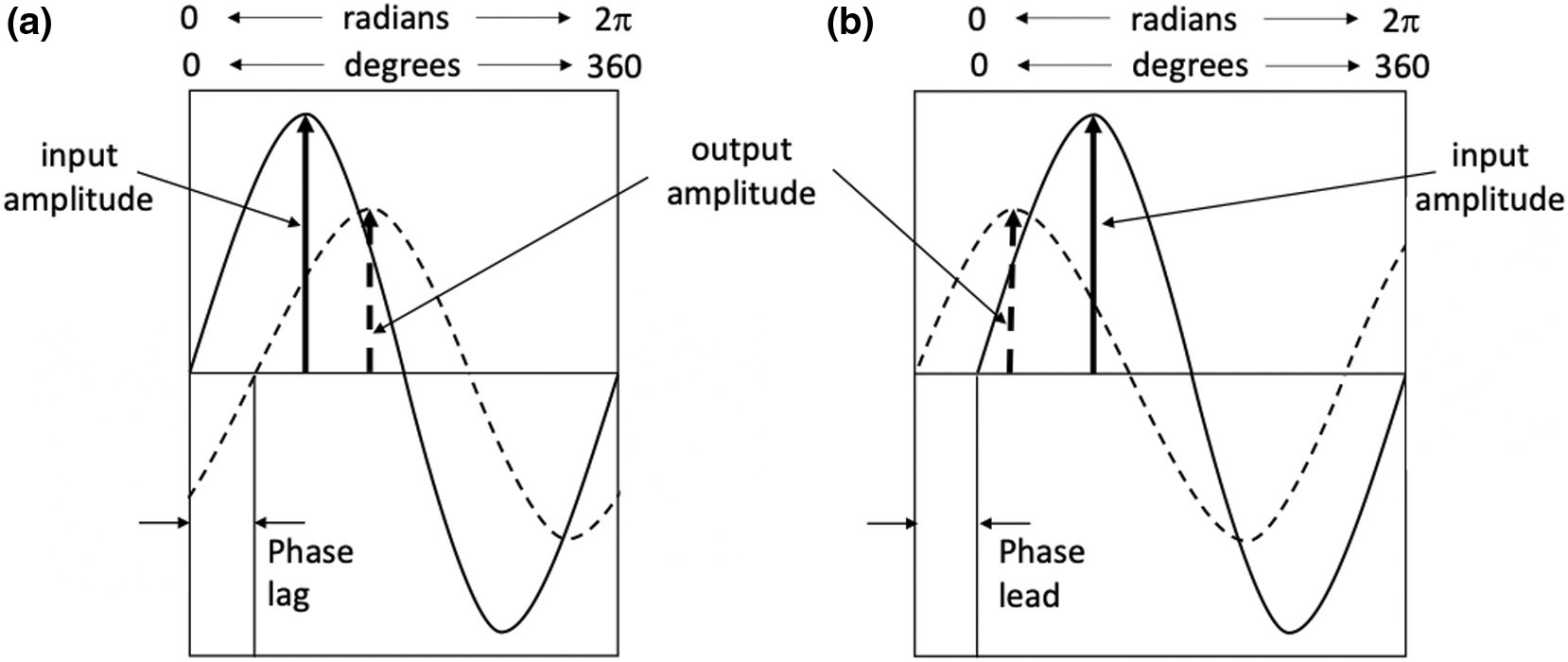
TFA provides the gain, phase, and coherence metrics for all frequencies and this illustrates one frequency. Gain is output amplitude / input amplitude and phase is the difference in the timing of the sine waves. (a) The output is positive and lags the input; phase is negative. (b) the output is negative and leads the input; phase is positive.

Transfer function analysis is a commonly used frequency analysis technique for investigating the temporal characteristics of a response to a stimulus (McKay et al., 1999), and can be used as a method of analyzing the BOLD response to a hypercapnic stimulus (Duffin et al., 2015). The relation between the BOLD response signal and the hypercapnic stimulus signal is analyzed in the frequency domain, resolving the two signals into their Fourier series of component sine waves. As a result, the magnitude (gain) of a response to a stimulus is calculated independently of any temporal offset (phase) between the measured waveforms. Gain measures the amplitude relation between the stimulus signal and the response signal. The phase difference between the stimulus and its response arises from two factors: A blood transit time delay and a vascular response time (Blockley et al., 2011). Coherence yields a measure of the constancy of the gain over time. TFA therefore offers a means of characterizing the BOLD response to CO_2_ that goes beyond an estimate of the magnitude of the response, as obtained from CVR, to provide insight into the response dynamics. TFA is calculated for all frequencies of the signal, but for the relation between the BOLD response and the CO_2_ stimulus a frequency of 0.01 Hz is chosen since its coherence is greatest at that frequency. Figure 1 illustrates the gain and phase metrics.

TFA gain, which is a metric like CVR, indicates the ability of the cerebrovascular regulatory mechanisms to respond to a stimulus, in this case CO_2_. In other words, it is the sensitivity of the regulator, and should be similar to that found in a healthy population; a decrease indicates the possibility of a poor response to a demand for flow with neural activity and possible resulting in tissue hypoxia. TFA phase, which is a metric like tau, indicates the speed of response to a stimulus, and should be as found in a healthy population. An increased phase or tau compared to a healthy group indicates a slowing of the vascular response to a vasodilatory stimulus. A slow response means that when an increase in flow is demanded, such as with neural activity, the required oxygen delivery is delayed, so there is brief tissue hypoxia.

Here we use a standardized vasoactive stimulus applied to a cohort of adult patients with SCD to measure their ability to vasodilate and the speed of this response. We hypothesize that both aspects are compromised in SCD patients. To test this hypothesis, we measured the CVR and speed of response in the time domain and confirmed these finding using the corresponding TFA measures gain and phase.

## 2 MATERIALS AND METHODS

### 2.1 Participant and ethics approval

This study conformed to the standards set by the latest revision of the Declaration of Helsinki and was approved by the Research Ethics Board of the University Health Network (UHN) and Health Canada. The reference number for this study is CAPCR 13–7168. All participants provided written and informed consent to partake in this study. The outpatient hematology clinic at UHN recruited 35 patients with SCD, referred to as SCD patient group over a 3‐year time frame (2018–2021). In addition, we previously recruited 24 healthy control volunteers by word of mouth, referred to as the HC. The HC group was non‐smokers, not on any medication and had no known history of neurological or cardiovascular disease. All participants were examined by a neuroradiologist (DJM) after scan acquisition for white matter hyperintensities (WHM) and strokes to ensure the selection of participants with no such complications. Participants with such complications were not included in the study.

### 2.2 Vasodilatory stimulus

**FIGURE 2:**
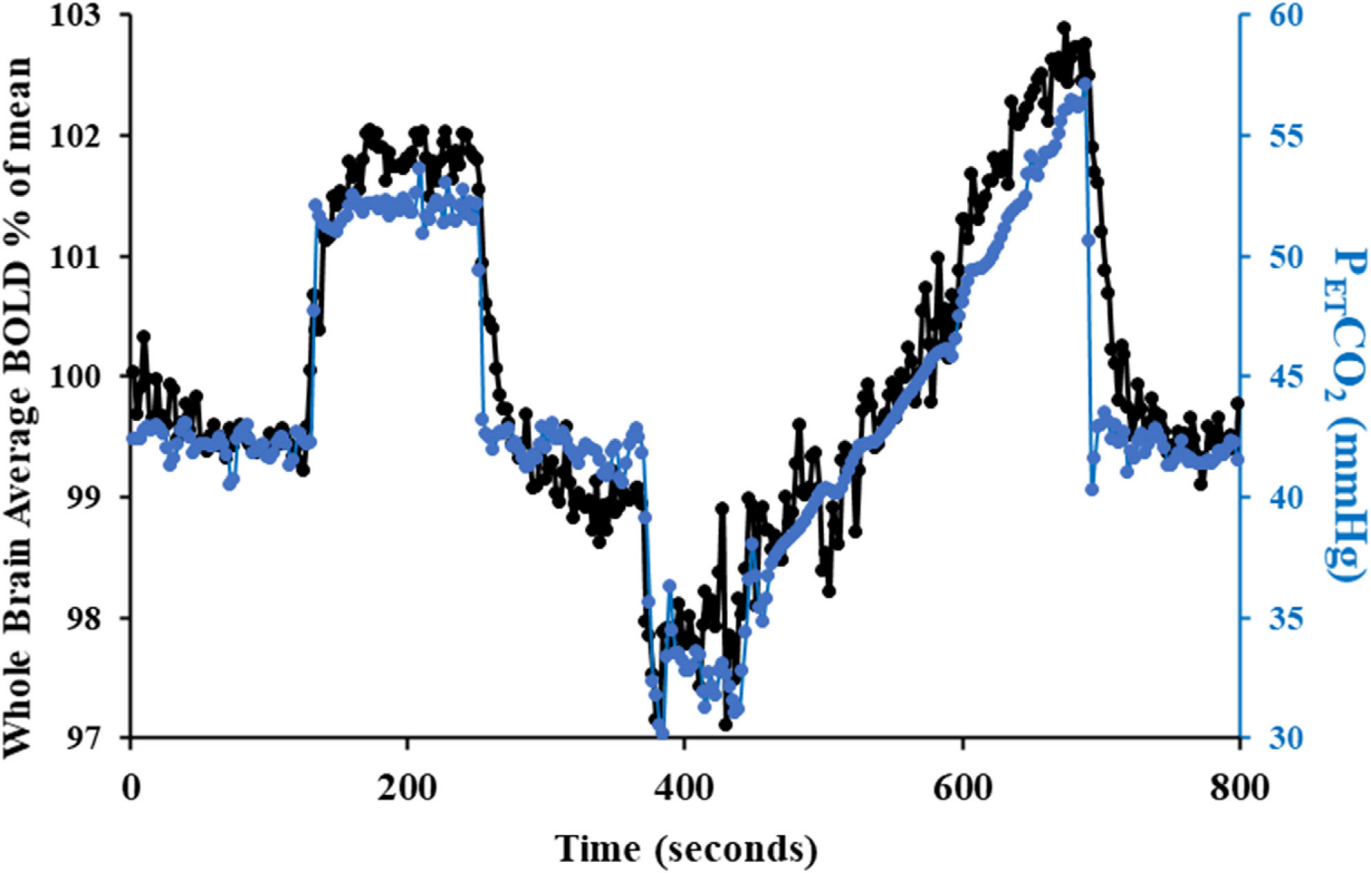
The CVR protocol implemented on a representative subject. The end tidal PCO_2_ stimulus (blue circles), and whole brain average BOLD response (black circles) are shown. The TFA calculates gain, phase, and coherence at the frequency of 0.01 Hz, symmetrical to resting P_ET_CO_2,_ so its gain and phase are relative to the resting values of PetCO_2_.

All participants underwent a standardized blood gas sequence protocol implemented by a computerized gas blender implementing prospective gas targeting algorithm whose targeting is independent of breathing pattern (Fisher et al., 2016; Slessarev et al., 2007) (RespirAct™, Thornhill Medical). The vasoactive stimulation protocol consisted of clamping end‐tidal partial pressure of CO_2_ (PetCO_2_) at subjects respective resting level for 2 min, a step increase of 10 mm Hg for 2 min and followed by a return to the subject's baseline for another 2 min. Next, the PetCO_2_ was reduced by 10 mm Hg from the subjects resting for 1 minute, followed by a steady rise (ramp) to 15 mm Hg above baseline over a 4.5‐min period, and returned to baseline for 2 more minutes (Figure 2).

### 2.3 MRI acquisition

The images acquired from both the HC and SCD patient groups were identical. The CVR scan was acquired on a 3‐Tesla GE scanner (HDx Signa platform, GE healthcare) with an 8‐channel head coil at Toronto Western Hospital. All participants completed an anatomical scan followed by a BOLD sequence scan. In addition, the SCD patient group was subject to further clinical scans as requested by their physicians including axial T2 flair, 3D ASL, DWI and EPI GRE. First, a whole‐brain coverage high‐resolution T1‐weighted 3D spoiled gradient echo sequence (anatomical images) was acquired with the following parameters: TI = 450 ms, TR = 7.88 ms, TE = 3 ms, flip angle = 12°, voxel size = 0.859 × 0.859 × 1 mm, matrix size = 256 × 256, 146 slices, field of view = 24 × 24 cm and no interslice gap. Second, a BOLD fMRI T2*‐weighted echoplanar imaging gradient echo sequence was attained with the following parameters: TR = 2400 ms, TE = 30 ms, flip angle = 85^°^, 41 slices, voxel size = 3.5x3.5 mm, matrix size = 64 × 64, number of frames = 335, field of view = 24 × 24 cm.

### 2.4 Data analysis

The acquired BOLD images were volume registered, slice‐time corrected and co‐registered to the anatomical images using AFNI software (National Institutes of Health) (Cox, 1996). The PetCO_2_ data were time‐shifted to the point of maximum correlation with the whole brain average BOLD signal such that it aligns with the rapid changes. A linear regression fit of the BOLD signal data to the PetCO_2_ data for the ramp portion was then performed on a voxel‐by‐voxel basis, and the slope taken as CVR. CVR was expressed as the percent change in BOLD signal per change in PetCO_2_ (%/mm Hg) (Fierstra et al., 2010; McKetton et al., 2018b). The speed of response (tau) was calculated from the step portion of the CVR protocol described in further detail elsewhere (Poublanc et al., 2015). Analytical processing software, SPM8 (Wellcome Department of Imaging Neuroscience, Institute of Neurology, University College), was used to segment the anatomical images (T1 weighted) into gray matter (GM) and white matter (WM) and a threshold of 70% probability was applied. This was done by re‐registering all files to the T1 MNI template (MNI152) via a nonlinear transformation (Ashburner & Friston, 1997) and spatially smoothed with a full width at half maximum of 5 mm, to minimize intersubject co‐registration errors. Then using SPM8 normalization, images were transformed into Montreal Neurological Institute (MNI) space, which uses a non‐linear re‐registration.

**FIGURE 3:**
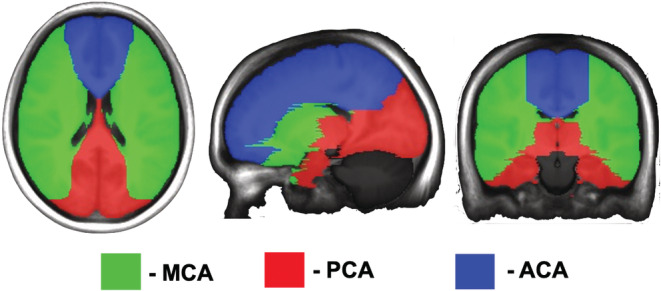
The axial, sagittal, and coronal view of the manually delineated regions of interest (ROI): MCA, PCA, ACA templates.

A custom program (LabVIEW, National Instruments, Texas) was used to calculate TFA parameters from the entire CVR protocol for each voxel. The alignment of PetCO_2_ with the BOLD (%) signal was adjusted to obtain the maximum average gain (Duffin et al., 2015). TFA maps were generated in original space and converted into MNI space. The CVR, tau, and TFA values were calculated in the WM, GM, and three regions of interest: middle cerebral artery (MCA), anterior cerebral artery (ACA), and posterior cerebral artery (PCA). The ROIs were manually delineated on an anatomical MNI template (Figure 3).

The 24‐person cohort of HCs was used to create an atlas to establish normative data for CVR, tau, and TFA parameters. Atlas creation involved co‐registering the maps into a standard space and calculating a voxel‐by‐voxel mean and standard deviation for each metric for each corresponding atlas. Atlas creation is discussed in further detail elsewhere (Poublanc et al., 2015; Sobczyk et al., 2015; McKetton et al., 2018a; Sobczyk et al., 2021). Results from a 35‐person SCD patient group cohort was compared to the healthy 24‐person HC atlas. The participant‐specific GM and WM masks generated previously were used to calculate GM and WM values for TFA, CVR, and tau parameters.

### 2.5 Statistical analysis

All CVR, tau, and TFA parameters in the HC and SCD patient groups were compared using a three way analysis of variance (ANOVA) with factors tissue (WM or GM), region of interest (MCA, PCA, ACA), and group (HC and SCD patient groups) using a commercial statistical package (SigmaPlot, Systat Software). Both Normality Test (Shapiro–Wilk) and Equal Variance Tests were part of the ANOVA, and correction for multiple comparisons were applied by an all pairwise multiple comparison procedure (Holm‐Sidak method). Significant difference was taken as *p* < 0.05. Using AFNI, a voxel‐wise mean difference between the healthy control and SCD patient group atlases were calculated for the CVR, tau, and TFA parameters to visualize areas of difference.

## 3 RESULTS

### 3.1 Participant characteristics

The participant demographics are summarized in Table 1. The SCD patient group consisted of 35 patients (19F) ranging from 18 to 74 (mean [SD] 32.1 ± 13.4, median age 28, IQR = 17). The HC group consisted of 24 participants (8F) ranging from 22 to 82 (mean [SD] 35.1 ± 13.8 years, median age 30, IQR = 15.5). None of the patients with SCD have previously experienced transfusion therapy. Some patients were treated with varying doses of Hydroxyurea (150–2000 mg), Vitamin D, Folic Acid, and various medications for pain management. All patient characteristics are found in Table S1.

**TABLE 1:** Summary of participant demographics

	HC group	SCD patient group
Age range		
18–28	10	17
29–38	6	10
39–54	6	5
55–83	2	3
Mean age (SD)	35.1 (13.8)	32.1 (13.4)
Sex		
F	8	19
M	16	16
Total	24	35

### 3.2 Atlas of CVR, Tau, and TFA

**FIGURE 4:**
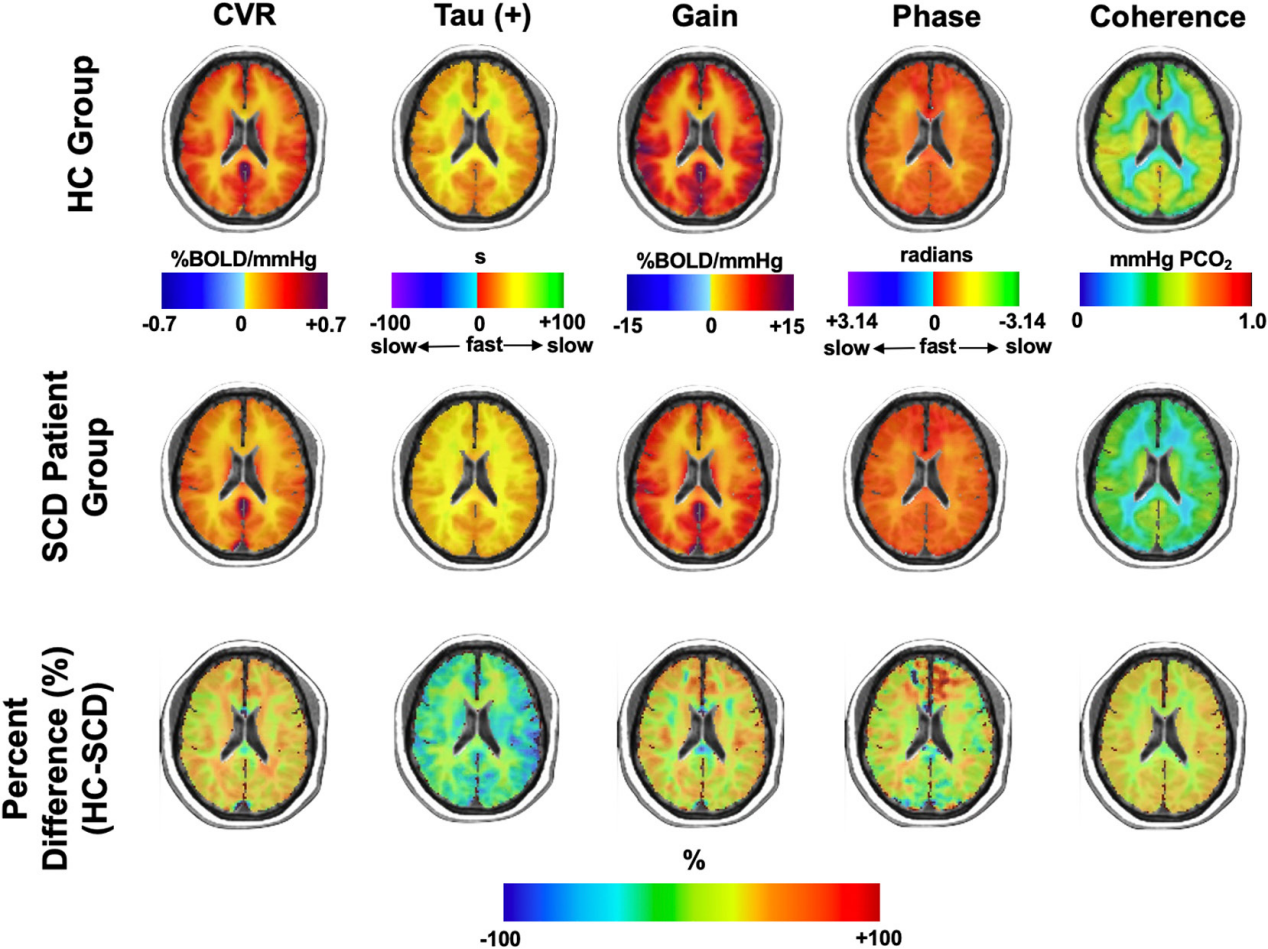
The average axial images of CVR, tau, and TFA parameters generated from the HC and SCD patient groups and the percent difference (%) calculation showing the areas of difference between the two groups.

The collective averaged maps of the HC and SCD patient groups are shown in Figure 4. The creation of an atlas where there is a collective average of maps is way of visually representing the group averages and a voxel wise comparison of percent difference (%) can be observed between the two groups. In all three regions for both GM and WM, the CVR, Gain and coherence are observed to be higher in the HC group in comparison to the SCD group. The tau and TFA parameter phase in all three regions for GM and WM is observed to be lower in the HC group in comparison to the SCD group. These observations of mean difference hold to be statistically significant as compared by a three‐way ANOVA (Table 2).

**TABLE 2:** A statistical summary of *p*‐values for the comparison of the HC and SCD patient groups in the white matter (WM) and gray matter (GM) for the middle cerebral artery (MCA), anterior cerebral artery (ACA), and posterior cerebral artery (PCA)

Interaction	CVR	Tau	Phase	Coherence	Gain
SCD/HC & WM/GM	<0.001	0.087	0.156	<0.001	<0.001
SCD/HC & MCA/ACA/PCA	0.082	0.767	0.486	0.848	0.98
WM/GM & MCA/ACA/PCA	0.004	0.004	0.011	0.011	0.992

	WM	GM	WM	GM	WM	GM	WM	GM	WM	GM
HC vs. SCD	<0.001	<0.001	<0.001	<0.001	<0.001	<0.001	<0.001	<0.001	0.015	<0.001
PCA vs. ACA	<0.001	<0.001	<0.001	0.892	<0.001	0.014	0.007	0.289	<0.001	<0.001
PCA vs. MCA	<0.001	<0.001	<0.001	0.786	<0.001	0.056	0.02	0.281	<0.001	<0.001
ACA vs. MCA	<0.001	0.135	0.412	0.984	0.334	0.521	0.632	0.038	<0.001	<0.001

### 3.3 CVR, Tau, and TFA parameters in ROIs

The three‐way ANOVA showed that all three factors were significant determinants of all the measures with *p* < 0.001 except coherence (*p* = 0.018). WM/GM is a significant determinant of the measures but does not depend on which territory or group and so no multiple comparison was done. Territory (MCA/ACA/PCA) is also a significant determinant, and in this case, significance depends on the other factors and so to isolate which group(s) differed from the others a multiple comparison procedure would be necessary (not reported). Finally, the effect of different levels of SCD/HC depends on what level of WM/GM is present. There is a statistically significant interaction between SCD/HC and WM/GM (*p* ≤ 0.001). However, the effect of different levels of SCD/HC does not depend on what level of MCA/ACA/PCA is present. There is not a statistically significant interaction between SCD/HC and MCA/ACA/PCA (*p* = 0.980). Consequently, a multiple comparison procedure was unnecessary for the SCD/HC within territories. A statistical summary of reported p‐values is displayed in Table 2. The effects sizes, calculated as h^2^ (%), and confidence intervals for the three‐way ANOVA are shown in Table 3.

**TABLE 3:** The effects size η^2^ (%) and confidence intervals for the three‐way ANOVA

Effect size η^2^ (%)					
Factor	CVR	Tau	Gain	Phase	Coherence
SCD/HC	7.6	5.1	6.3	3.3	14.1
WM/GM	54.5	36.0	57.4	11.4	28.1
MCA/ACA/PCA	17.9	3.0	8.0	0.5	1.3

Confidence interval			
		Upper	Lower
SCD			
CVR	WM	0.1064	0.0844
	GM	0.2181	0.1852
Tau	WM	46.3764	39.9906
	GM	31.9366	27.1779
Phase	WM	−0.4519	−0.5769
	GM	−0.3060	−0.3866
Coherence	WM	0.3624	0.3109
	GM	0.4556	0.3855
Gain	WM	3.1053	2.4912
	GM	6.5694	5.3515
HC			
CVR	WM	0.1262	0.1096
	GM	0.2871	0.2596
Tau	WM	42.9363	36.4809
	GM	25.0673	19.3231
Phase	WM	−0.5649	−0.676
	GM	−0.3390	−0.4231
Coherence	WM	0.4002	0.3576
	GM	0.5768	0.5192
Gain	WM	3.4945	2.9560
	GM	8.6009	7.7371

**FIGURE 5:**
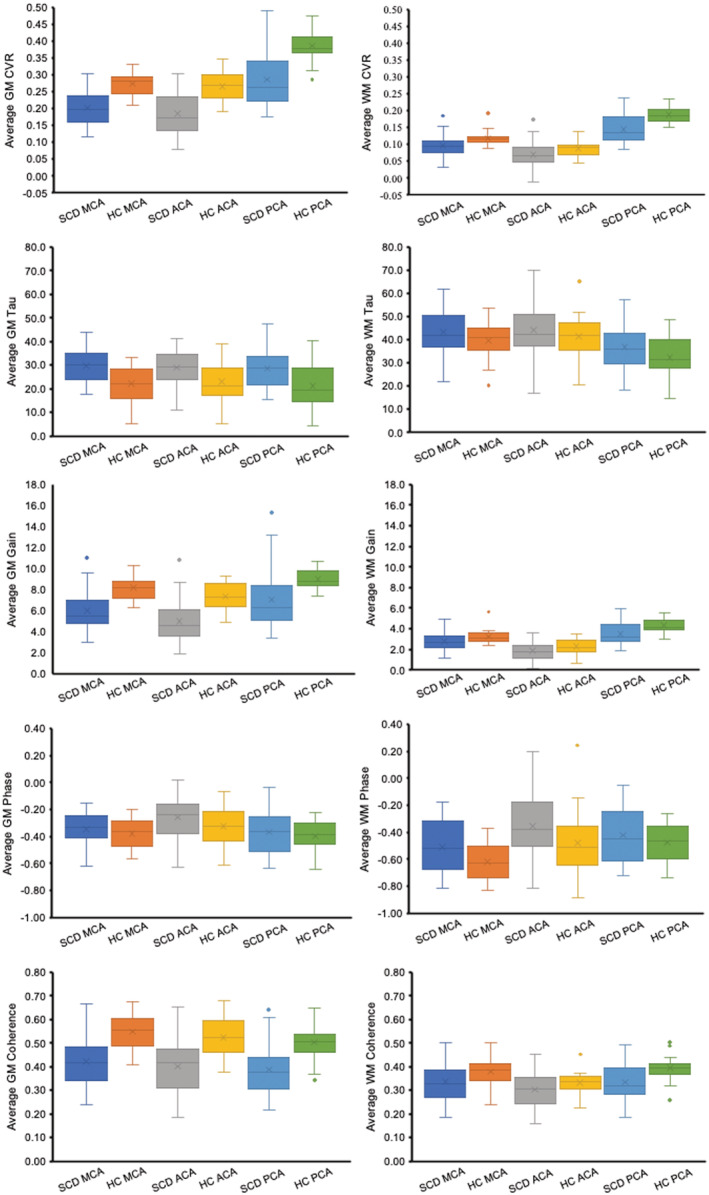
Boxplots showing the distribution of the metrics for the three ROI: MCA, ACA, and PCA in both the SCD and HC group.

The CVR, tau, and TFA parameters are presented in Table 4 for WM and Table 5 for GM since region (WM/GM) was significantly different for all regions of interest. The lower CVR and gain was detected in the GM and WM of SCD patient group in comparison to the HC group (*p* < 0.001) for all regions. Tau and phase, measures of speed of response, were concordant, with both metrics, prolonged in SCD in WM and GM in all regions. Coherence was lower in the SCD patient group in all regions. Figure 5 presents the distribution of the data using boxplots.

**TABLE 4:** The average (SD) WM CVR, tau and TFA parameters in the HC and SCD patient groups for the middle cerebral artery (MCA), anterior cerebral artery (ACA), and posterior cerebral artery (PCA)

	MCA		ACA		PCA	
	HC Group	SCD Group	HC Group	SCD Group	HC Group	SCD Group
CVR (%/mm Hg)	0.118 (0.021)	0.095 (0.033)	0.088 (0.023)	0.069 (0.036)	0.187 (0.023)	0.144 (0.04)
Tau (s)	39.709 (8.068)	43.184 (9.499)	41.397 (8.864)	44.163 (10.939)	32.178 (8.594)	36.957 (9.727)
Gain (%/mm Hg)	3.225 (0.673)	2.798 (0.913)	2.241 (0.711)	1.833 (0.849)	4.284 (0.573)	3.508 (1.118)
Phase (radians)	−0.62 (0.138)	−0.514 (0.186)	−0.483 (0.248)	−0.354 (0.238)	−0.478 (0.14)	−0.422 (0.206)
Coherence	0.379 (0.053)	0.337 (0.077)	0.333 (0.043)	0.303 (0.073)	0.395 (0.06)	0.335 (0.076)

*Note*: All metrics in all ROIs were significantly different between HC and SCD patient groups (three‐way ANOVA, *p* < 0.001).

**TABLE 5:** The average (SD) GM CVR, tau, and TFA parameters in the HC and SCD patient groups for the middle cerebral artery (MCA), anterior cerebral artery (ACA), and posterior cerebral artery (PCA)

	MCA		ACA		PCA	
	HC Group	SCD Group	HC Group	SCD Group	HC Group	SCD Group
CVR (%/mm Hg)	0.273 (0.035)	0.202 (0.049)	0.265 (0.042)	0.185 (0.059)	0.386 (0.047)	0.286 (0.075)
Tau (s)	22.195 (7.179)	29.557 (7.079)	22.905 (7.795)	28.913 (7.637)	21.218 (8.362)	28.527 (8.042)
Gain (%/mm Hg)	8.169 (1.08)	5.96 (1.812)	7.317 (1.332)	4.938 (2.062)	8.962 (0.848)	7.022 (2.623)
Phase (radians)	−0.381 (0.105)	−0.346 (0.12)	−0.326 (0.147)	−0.258 (0.159)	−0.4 (0.116)	−0.369 (0.159)
Coherence	0.548 (0.072)	0.421 (0.104)	0.523 (0.077)	0.402 (0.11)	0.503 (0.079)	0.388 (0.1)

*Note*: All metrics in all ROIs were significantly different between HC and SCD patient groups (three‐way ANOVA, *p* < 0.001).

### 3.4 CVR, Tau, and TFA maps

**FIGURE 6:**
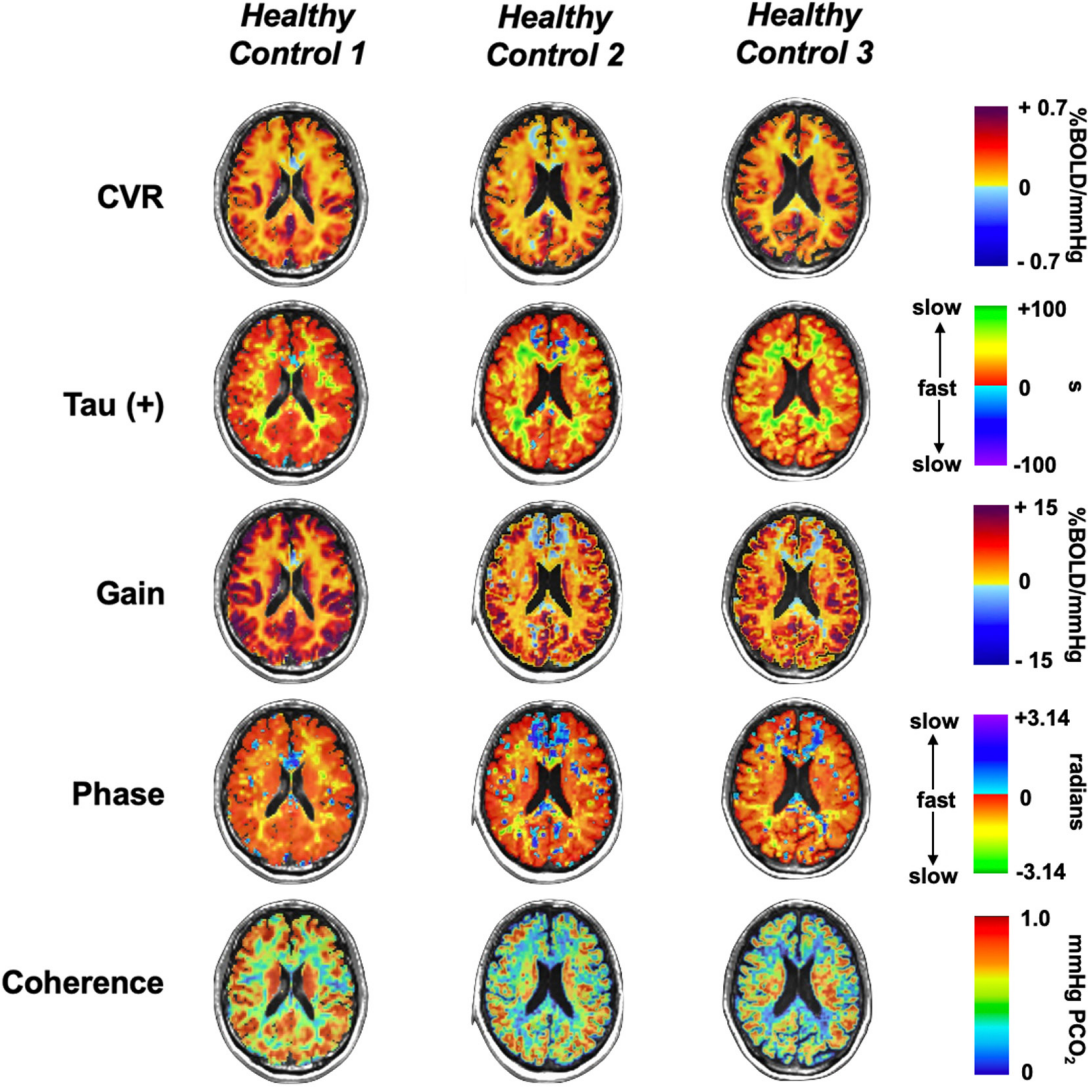
Single axial slices for 3 representative healthy controls for CVR, tau, and the three TFA parameters are displayed.

**FIGURE 7:**
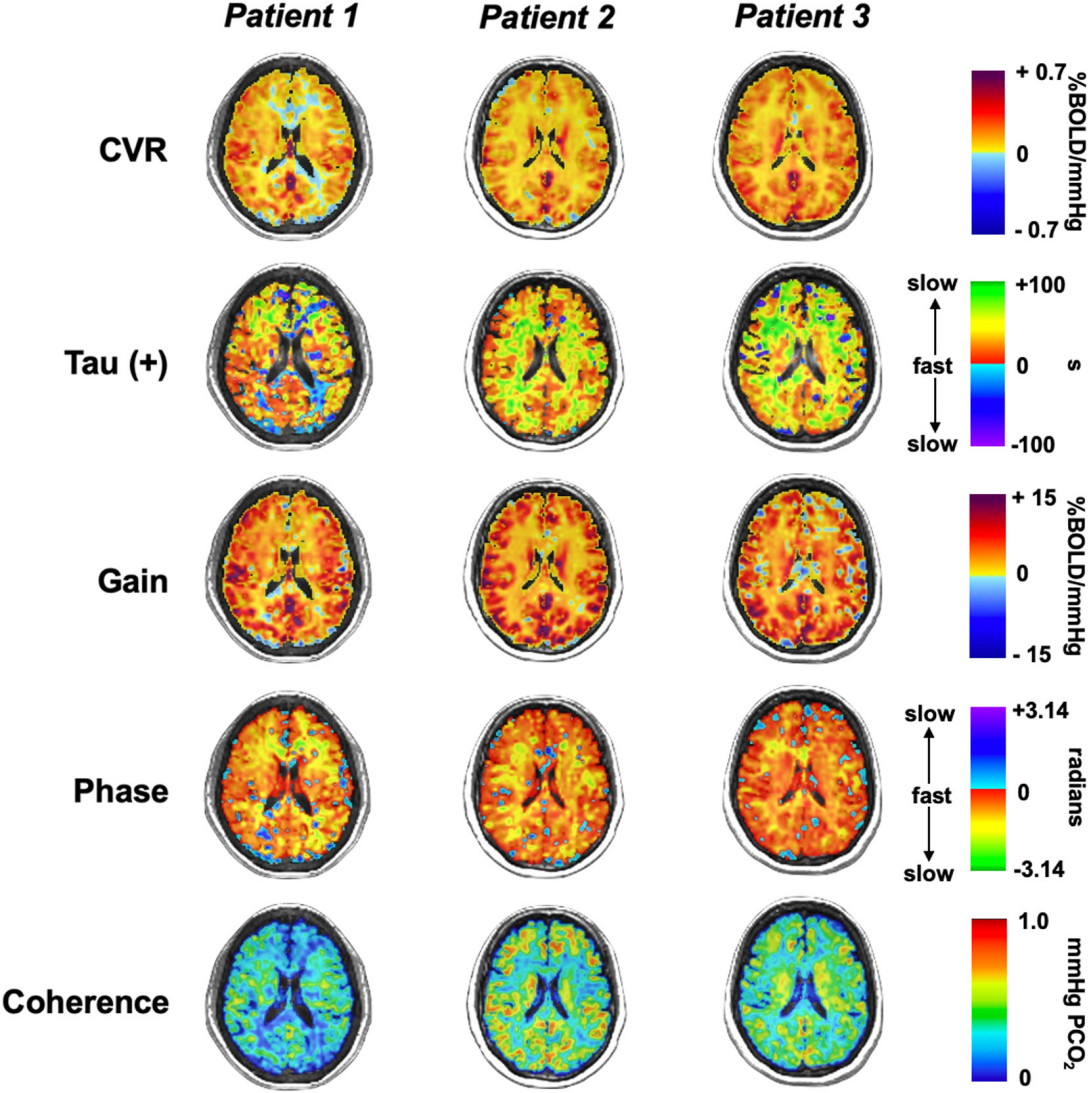
Single axial slices for 3 representative patients with SCD for CVR, tau, and the three TFA parameters are displayed.

Figures 6 and 7 show a single axial slice for 3 representative healthy controls and patients, respectively, with SCD for CVR, tau and TFA parameters, gain, phase, and coherence. The variability in the degree of abnormality within patients and across the 5 metrics can be observed in comparison to the healthy control examples. The overall reduction in CVR, TFA gain, and TFA coherence can be noted with an increase in tau and TFA phase metrics in all three patient examples. However, there is small variability within the patient population due to differences in sex, age, and progression of disease.

## 4 DISCUSSION

In this study we document the vascular responses to a vasoactive stimulus in adults with SCD, compared to healthy adults, using CVR analysis (Sobczyk et al., 2015) and TFA (Leung, Duffin, et al., 2016). The main findings are that in patients with SCD, CVR, gain and coherence are reduced in the ROIs while tau and phase are increased, indicating a decreased and slower response. These findings confirm the observations of previous studies that have noted reductions in vasodilatory reserve in patients with SCD (Sayin et al., 2022) and pediatric patients with SCD (Kosinski et al., 2017; Leung, Kosinski, et al., 2016). Here we report the first TFA assessment in adult sickle cell individuals, calculating the CVR, tau, and TFA parameters for an overall group comparison.

Previously, we explored CVR and resistance parameters in patients with SCD (Sayin et al., 2022). We studied a similar cohort of SCD and healthy control individuals but wanted to explore other parameters that could aid in further understanding the underlying pathophysiology of this disease in adult patients with SCD. Resistance is made as a steady state measurement where the sigmoid response to a vasoactive stimulus is measured. We found that cerebrovascular reserve is reduced in patients with SCD compared to healthy controls as evidenced by decreased CVR and decreased resistance reserve at resting PetCO_2_. The extent to which cerebrovascular resistance can be adjusted is also compromised, with a reduced resistance sigmoid amplitude. However, the mechanism of adaptation to a generally higher resting PetCO_2_ appears similar to that of healthy controls as shown by the increase in resistance sigmoid midpoint. Whereas our current findings measure CVR (gain) and provide information on the speed of response (phase).

Leung, Duffin, et al. (2016) concluded that CVR and TFA gain was decreased in WM and GM in pediatric patients with SCD compared to their age‐matched controls. Leung, Kosinski, et al. (2016) studied children (10–18 y) with TFA, but the experiments used a different CO_2_ stimulus consisting of a series of short increases in PetCO_2_ from 40 to 45 mm Hg. The fixed baseline of 40 mm Hg contrasts with that used here, which was fixed to the subject's resting PetCO_2_. They found that gain and CVR was reduced in agreement with our findings. However, phase lag was not different from healthy controls in contrast to the increased phase lag found in these experiments. Whether this difference was due to the adult versus children aspect or the different stimulus paradigms is not determinable. However, in continuation of Leung, Duffin, et al. (2016) our findings demonstrate that with adulthood, the progression of disease is still present and does not improve.

Other previous studies have sought to assess adequacy of perfusion at rest by measuring conditions that may result in cerebral ischemia such as low CBF (Fantini et al., 2016), or the presence of ischemia such as measures of increased OEF (Fields et al., 2018). However, oxygen extraction fractions in patients with SCD are mixed in this area, with some studies indicating normal OEF (Bush et al., 2014; Li et al., 2020), and others indicating higher (Fields et al., 2018; Jordan et al., 2016)and lower OEF (Vaclavu et al., 2020). Surrogate measures of flow such as TCD‐measured MCAv do not adequately convey regional differences in CBF beyond the area of stenosis (Fukushima et al., 1999).

Our aim in this study was to gather more information regarding the dynamic responses of cerebral vasculature of patients with SCD that may account for the reduced effective oxygen delivery to the cortex and watershed areas of the brain. Such investigations must include a repeatable vasoactive stimulus within a group and between groups, and the performance of a broad survey of vascular responses. We applied a highly repeatable degree and pattern of isoxic hypercapnia and measured a series of flow parameters via CVR and TFA analysis to assess flow response. The uniformity of the stimulus canceled its effect as a source of variability and enabled the characterization of responses within each group as representing the underlying physiology. We report a number of differences in vascular response in patients with SCD compared to HC group, but it is not clear which of these are related only to anemia and which are part of SCD. In patients with SCD, the vascular response is impaired in comparison to a healthy population. This is seen in reduced CVR and gain in the MCA, ACA, and PCA regions. The responsiveness of these major vessels to a vasoactive stimulus like carbon dioxide is clearly delayed. This delay, also known as the speed of response (in seconds) is longer in patients with SCD. These differences are therefore widespread and not confined to specific regions, indicating that the vessel pathology is a general condition of SCD.

In this study we used a CO_2_ challenge as the vasoactive stimulus (Fierstra et al., 2013) and the BOLD signal changes as a surrogate for CBF. The change in the BOLD response is relative to the magnitude of change in PaCO_2_ and was therefore normalized as %BOLD/mm Hg delta PCO_2_ (Mandell et al., 2008). This CVR metric is decreased in individuals with SCD (Kim et al., 2016; Kosinski et al., 2017; Leung, Kosinski, et al., 2016; Nur et al., 2009), indicating a decreased ability to increase CBF in response to a vasodilatory stress, suggesting that vessel diameter has approached its maximum. Our results also support this finding that the SCD patient group has a lower CVR in the MCA, ACA, and PCA regions for both WM and GM tissue.

TFA has previously been used to examine cerebrovascular pressure autoregulation (Blaber et al., 1997; Tzeng et al., 2012; Zhang et al., 1998), and is an alternative method of analyzing the BOLD response to CO_2_ (Duffin et al., 2015). It offers additional insights to cerebrovascular function in that it measures the response dynamics (phase) as well as the magnitude of the BOLD response to CO_2_ (gain) and the coherence. Rather than a linear regression of the BOLD versus CO_2_ as in CVR, gain measures the power transferred between the stimulus signal and the response. The phase difference of the stimulus and its response comprises two factors: a blood transit time delay and a vascular response time (Blockley et al., 2011) also measured as tau, an index of the speed of response (Poublanc et al., 2015; Poulin et al., 1996). Coherence yields a measure of the constancy of the gain over time. Indeed, the physiological interpretation of TFA metrics can be viewed as the same as CVR and tau, as it is only a different technique of measuring the response.

TFA is therefore an alternative method of analyzing the BOLD response to CO_2_, and as expected the results demonstrate a complementary outcome between these two methods (Tables 2 and 3). Similar to the CVR metric, the gain was also reduced in patients with SCD compared to HC group. The phase and tau measurements showed that the speed of response was reduced in the SCD group. We suggest that the lower CVR and gain metric in SCD patients compared to the HC group show that the regulatory abilities of the SCD group cerebrovasculature is likely to be insufficient to maintain the CBF supply when it is compromised due to the development of further pathology. Furthermore, SCD patients with increased tau or phase metric in comparison to the HC group means that the speed of response is slower, which also indicates a regulatory insufficiency.

This study measured cerebrovascular function in adults using BOLD MRI, whereas clinical assessments rely heavily on TCD measurements. In children with SCD the risk of stroke can be stratified with transcranial doppler (TCD) velocity measurements of the MCA (Adams et al., 1992; Adams et al., 1998; Adams & Brambilla, 2005; Ware et al., 2016) and (National Institutes of Health, 2014). However, the use of TCD as a tool in stroke risk assessment is entirely confined to children with SCD. The use TCD in adults is more difficult because of the closure of the bone window (Naqvi et al., 2013), and its prediction of stroke is less persuasive. Limited evidence suggests that TCD of the MCA is lower in adults compared to children and associated with intracranial stenosis (Silva et al., 2009), but there is no evidence to suggest that elevated TCD can predict future infarcts in adults with SCD. The TCD cut‐off of 200 cm/s, above which children with SCD are placed on regular transfusion for primary stroke prophylaxis, is not applicable in adults.

Nonetheless, adults with SCD have high risk of ischemic and hemorrhagic stroke with high mortality rate (National Institutes of Health, 2014; Ohene‐Frempong et al., 1998). Silent cerebral infarcts (SCI) are more in prevalence in adults with SCD (Houwing et al., 2020), with a high risk of recurrence (Houwing et al., 2020; Jordan et al., 2018) and neurocognitive impairment. However, specific indications for the use of neuroimaging studies like CTA, MRI, and MRA in adults with SCD are controversial (National Institutes of Health, 2014), with little evidence to guide their use. Consequently, this study is intended to provided evidence in support of the use of MRI to assess cerebrovascular health in adults. These findings endorse the importance of MRI screening for adults with Hb SS or Hb S‐Beta‐thalassemia as recommended by the American Society of Hematology.

A future direction of these studies will be aimed at identifying TFA biomarkers for SCI and overt stroke risk in adults with SCD, and as a tool to evaluate the efficacy of existing and novel SCD modifiers in improving cerebral vascular tone.

### 4.1 Limitations

A previous study of CVR in healthy individuals found no significant changes in CVR were detected until extreme age (McKetton et al., 2018a). We assume that the same would be the case for TFA metrics and this suggest that age difference between the HC and SCD groups in this study is unlikely to account for our findings. Nevertheless, differences in sex, age, and race within our SCD patient population and between the HC and SCD groups impose a degree of uncontrolled variability in this small cohort, and consequently may have contributed to the differences we found. Sample size was determined by the number of clinical patients available to us during the 3‐year time frame with our exclusion criteria.

The measures of CBF are based on BOLD measurements, which provide a convenient surrogate of CBF. However, the BOLD signal also reflects the influence of other factors such as cerebral blood volume. Nevertheless, BOLD has been shown to be a reliable surrogate for CBF in patients (Mandell et al., 2008) and we suggest that until shown to be different between patients and controls we accept BOLD as a reasonable surrogate for CBF. Detailed discussions of the appropriateness of BOLD as a flow estimate have been provided in both Duffin et al. (2015) and Tancredi et al. (2015). Here we present only comparisons between the two groups, but evaluations may be made for individual SCD patients by comparing them to the atlas of the healthy control group and computing z‐scores as described in Sobczyk et al. (2015).

## 5 CONCLUSIONS

We used CVR analysis and TFA to explore the vascular response to a vasoactive stimulus in adult patients with SCD and compared the data to that from a healthy adult cohort to identify the degree of discrepancy from the normal range. Our data are in accord with previous observations of a reduction from the normal range of vasodilatory reserve in patients with SCD. Patients with SCD have a reduced CVR, TFA gain and coherence on average in all ROI. Furthermore, tau and TFA phase are increased (slowed response). Consequently, SCD patients are less able to increase CBF under stress, and the response is slower. These factors have a negative impact on the regulation of oxygen delivery.

## AUTHOR CONTRIBUTIONS

ESS, JAF, and JD designed the study. KHMK and DJM selected and reviewed all subjects for suitability. ESS, OS, and JP executed the experiments to acquire the data. ESS and JD analyzed the data and wrote the initial draft of the manuscript. All authors participated in the preparation and revision of the final version of the manuscript.

## CONFLICT OF INTEREST

JAF and DJM contributed to the development of the automated end‐tidal targeting device, RespirAct™ (Thornhill Research Inc., TRI) used in this study and have equity in the company. OS and JD receive salary support from TRI. TRI provided no other support for the study. All other authors have no disclosures to report.

## Supporting information


Table S1
