# Supplementary material for: Assessment of cerebrovascular function in patients with sickle cell disease using transfer function analysis
Source: Physiol Rep. 2022 Oct 5;10(19):e15472. doi: 10.14814/phy2.15472 (PMC9535348; doi:10.14814/phy2.15472)
Supplement: Supplementary file 1 — Table S1 [file PHY2-10-e15472-s001.docx]

| *Subject ID*  **Supplementary Table 1.** The characteristics of the patient group with sickle cell disease. | *Sex* | Hemoglobin  Pathology | *Hemoglobin (g/dL)* | *Hematocrit*  *(L/L)* | *Medication* |
| --- | --- | --- | --- | --- | --- |
| 1 | F | SC | 108 | 0.304 | Hydroxyurea |
| 2 | M | S/beta zero thalassemia | 108 | 0.32 | none |
| 3 | F | SS | 75 | 0.24 | Folic Acid, Vitamin D |
| 4 | M | SS | 106 | 0.29 | Hydroxyurea, Folic Acid, Vitamin D |
| 5 | M | SC | 137 | 0.37 | Indapamide, Amlodipine, Vitamin D |
| 6 | M | SS | 71 | 0.21 | Hydroxyurea, Vitamin D |
| 7 | F | SC | 108 | 0.299 | Amitriptyline, Gabapentin, Vitamin D |
| 8 | M | S/beta zero thalassemia | 80 | 0.25 | Hydroxyurea |
| 9 | F | SS | 95 | 0.27 | Hydroxyurea, Folic Acid |
| 10 | F | SS | 72 | 0.22 | Folic Acid, Vitamin D |
| 11 | M | SS | 110 | 0.35 | Xarelto |
| 12 | M | SS | 116 | 0.34 | Hydroxyurea, Statex |
| 13 | M | SS | 107 | 0.30 | Hydroxyurea, Vitamin D, Folic Acid |
| 14 | F | SS | 89 | 0.274 | Hydroxyurea |
| 15 | M | S/beta thalassemia | 107 | 0.33 | Adalet |
| 16 | F | SS | 113 | 0.32 | Folic Acid, Multivitamin |
| 17 | F | SC | 95 | 0.29 | None |
| 18 | M | SS | 93 | 0.26 | None |
| 19 | M | S/beta plus thalassemia | 118 | 0.36 | Hydroxyurea, Folic Acid |
| 20 | F | SC | 80 | 0.23 | Hydroxyurea, Folic Acid |
| 21 | M | SC | 125 | 0.350 | Hydroxyurea, Folic Acid |
| 22 | F | SS | 99 | 0.27 | Folic Acid |
| 23 | F | SC | 115 | 0.31 | Hydroxyurea, Vitamin D |
| 24 | F | SS | 96 | 0.28 | Vitamin D |
| 25 | F | SS | 68 | 0.189 | Hydroxyurea, Vitamin D |
| 26 | F | SS | 88 | 0.25 | Folic Acid |
| 27 | M | S/beta thalassemia | 108 | 0.34 | Vitamin D |
| 28 | F | SS | 86 | 0.25 | Hydroxyurea, Vitamin D, Folic Acid |
| 29 | M | SS | 102 | 0.30 | Hydroxyurea, Folic Acid |
| 30 | F | SS | 87 | 0.25 | None |
| 31 | F | SS | 89 | 0.27 | Vitamin D |
| 32 | F | SD | 93 | 0.27 | Vitamin D |
| 33 | F | SS | 85 | 0.25 | None |
| 34 | M | SS | 107 | 0.30 | Hydroxyurea, Vitamin D, Folic Acid |
| 35 | M | S/beta zero thalassemia | 107 | 0.31 | Hydroxyurea, Vitamin D |
